# Recent Progress in Research on Mechanisms of Action of Natural Products against Alzheimer’s Disease: Dietary Plant Polyphenols

**DOI:** 10.3390/ijms232213886

**Published:** 2022-11-11

**Authors:** Yi Wang, Kaiyue Wang, Junyuan Yan, Qian Zhou, Xiaoying Wang

**Affiliations:** 1 Key Laboratory of Pharmacology of Traditional Chinese Medical Formulae Ministry of Education, Tianjin University of Traditional Chinese Medicine, Tianjin 301617, China; 2 College of Traditional Chinese Medicine, Tianjin University of Traditional Chinese Medicine, Tianjin 301617, China

**Keywords:** Alzheimer’s disease, polyphenols, antioxidant, anti-neuroinflammation, amyloid-β, bioavailability

## Abstract

Alzheimer’s disease (AD) is an incurable degenerative disease of the central nervous system and the most common type of dementia in the elderly. Despite years of extensive research efforts, our understanding of the etiology and pathogenesis of AD is still highly limited. Nevertheless, several hypotheses related to risk factors for AD have been proposed. Moreover, plant-derived dietary polyphenols were also shown to exert protective effects against neurodegenerative diseases such as AD. In this review, we summarize the regulatory effects of the most well-known plant-derived dietary polyphenols on several AD-related molecular mechanisms, such as amelioration of oxidative stress injury, inhibition of aberrant glial cell activation to alleviate neuroinflammation, inhibition of the generation and promotion of the clearance of toxic amyloid-β (Aβ) plaques, inhibition of cholinesterase enzyme activity, and increase in acetylcholine levels in the brain. We also discuss the issue of bioavailability and the potential for improvement in this regard. This review is expected to encourage further research on the role of natural dietary plant polyphenols in the treatment of AD.

## 1. Introduction

Alzheimer’s disease (AD), also known as senile dementia, is a neurodegenerative disease that occurs in the elderly worldwide and develops very slowly. AD is the most common type of dementia [1]. AD is characterized by a variety of pathological features, including cerebral cortex atrophy, decrease in brain weight [2], loss of locus coeruleus neuromelanin [3], accumulation of extracellular amyloid-β protein (Aβ), and the occurrence of neurofibrillary tangles (NFTs) due to abnormal tau protein hyperphosphorylation [4]. The pathogenesis of AD remains unclear to date. Several hypotheses have been developed in the past, such as the oxidative stress [5], cholinergic injury hypothesis [6], Aβ toxicity hypothesis, tau protein phosphorylation hypothesis [7], neuroinflammation hypothesis [8], and apolipoprotein E (ApoE) gene hypotheses [9]. The complexity of these hypotheses is considered to be the main reason for the repeated failure of anti-AD drugs. Thus, no clear conclusions have been drawn to date regarding the factors that determine the occurrence and development of AD. Therefore, the clinical treatment of AD remains a challenge for mankind.

Despite the fact that AD was first discovered more than a hundred years ago and that major pharmaceutical companies such as Merck and AstraZeneca have made heavy investments in developing drugs against AD, the failure rate remains very high. Only six drugs have been approved by the Food and Drug Administration (FDA) for AD treatment to date; however, many adverse drug reactions have also been reported for these drugs [10] (Table 1).

Recently, the sodium oligomannate capsules (GV-971) for AD treatment were approved in China, which demonstrates the potential of natural products for the treatment of neurodegenerative diseases [23]. Therefore, the discovery and development of safe and effective anti-AD drugs from natural sources such as plants may provide a viable strategy [24]. Highly efficient anti-AD drugs with low toxicities may indeed be discovered through extensive studies on the effects of various natural products on AD-related molecular mechanisms [25].

**Figure 1:**
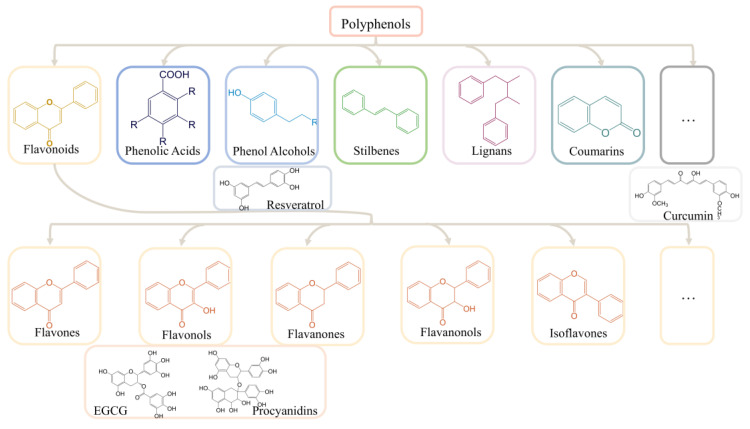
The classification of polyphenols. EGCG, ((−) -epigallocatechin-3-gallate).

The focus of AD treatment strategies is also gradually shifting to alternative therapies. A diet-based lifestyle is the most effective that can be easily adopted by individuals among these alternative therapies [26,27]. Improving the dietary structure, such as choosing a Mediterranean or Asian diet rich in fruits and vegetables, is recognized globally as an effective means to prevent the development of AD [28,29,30]. Polyphenols are commonly found in vegetables, fruits, and cereals such as grapes, blueberries, and apples, in addition to tea and red wine [31,32,33,34,35]. Polyphenols have diverse and complex structures. Most polyphenols are found in glycosidic forms in plants [36]. Polyphenols are mainly classified into flavonoids and nonflavonoids based on the basic flavonoid structure, aglycone. Flavonoids include flavones, flavonols, flavanones, flavanonols, and isoflavones, whereas nonflavonoids are mainly classified into phenolic acids, phenolic alcohols, stilbenes, lignans, curcumins, and coumarins [37] (Figure 1). Polyphenols have been widely used for the treatment of age-related neurological disorders, including AD and cognitive disorders, due to their antioxidant and anti-inflammatory activities [38]. Many in vivo and in vitro experiments have confirmed the therapeutic effect of polyphenols on neurodegenerative diseases, including AD. These experiments also clarified several AD-related molecular mechanisms and led to the development of several hypotheses regarding the development of AD, such as oxidative stress, cholinergic, Aβ toxicity, tau protein, and neuroinflammation hypothesis [39,40,41,42].

However, at present, most of the experiments on the application of polyphenols in the treatment of AD are limited to in vitro experiments or animal experiments, and there are few clinical trials in vivo human studies, which may be related to the low bioavailability in the brain and the difficulty in penetrating the blood–brain barrier (BBB) [43]. Meanwhile, the clinical trials that have been carried out are often limited by the small sample size, the short study duration, and the individual differences of the subjects, and cannot obtain encouraging results like in vitro experiments [44]. These issues need to be further considered in future clinical experiment design involving in vivo human studies.

As of 25th September 2022, we have selected the relevant studies from PubMed, CNKI, and Google Scholar databases, using the following Medical Subject Headings (MeSH) keywords: “polyphenols”, “plant polyphenols”, “Alzheimer’s disease”, “resveratrol”, “curcumin”, “procyanidins”, “EGCG”, “bioavailability”, and so on. We identified the relevant papers and selected them following the inclusion criteria: full-text original articles, including both preclinical and clinical studies.

Here, we review the regulatory roles of the most widely reported dietary plant polyphenols, curcumin, resveratrol, proanthocyanidins, and EGCG, on widely recognized molecular mechanism hypotheses related to AD development. We also discuss the current application limitations of polyphenols and improved sample preparation methods in order to provide a reference for further functional research on plant polyphenols for the prevention and treatment of AD. We also expect that this review will provide a theoretical basis for the development of similar health products and drugs.

## 2. Polyphenols and Oxidative Stress

### 2.1. The Oxidative Stress in Alzheimer’s Disease

The brain has a greater lipid content and energy requirements than any other organ. The brain also has relatively weak endogenous antioxidant defenses and is thus vulnerable to an imbalance in redox homeostasis [45]. Therefore, free radicals may readily disrupt neuronal cell structure and function and lead to various neurodegenerative diseases, including AD and Parkinson’s disease (PD) [46]. Oxidative stress (OS) has previously been shown to be the primary cause of neuronal death and neuroinflammation in neurodegenerative diseases [47]. The aggregation of misfolded proteins, such as amyloid fibers and aberrantly phosphorylated tau protein, is a major pathological feature of AD that disrupts normal mitochondrial function and induces the production of large amounts of reactive oxygen species (ROS). This, in turn, leads to the oxidization of Aβ scavenger proteins and thus to further Aβ accumulation, as well as to hyperphosphorylation and Tau protein aggregation [48,49,50].

The antioxidant enzyme system is the first line of defenses against ROS in the human body. Activities of glutathione peroxidase (GPx), glutathione reductase (GR), superoxide dismutase (SOD), and catalase (CAT) in the brains of AD patients have been reported to decrease, whereas malondialdehyde (MDA) activity is increased. This is accompanied by extensive protein and lipid peroxidation, which eventually leads to cell apoptosis [51]. SOD activity is particularly an important indicator of the degree of an oxidative stress injury in the nervous system. The regulation of SOD, GPX, GR, and CAT activities has thus become a focus of research for drug development against AD.

OS is also a significant feature of neuroinflammation and apoptosis. OS occurs in nerve cells; ROS produced by OS causes mitochondrial respiratory chain energy metabolism disorder, which leads to the irreversible opening of the mitochondrial permeability transition pore (MPTP) and a decrease in mitochondrial membrane potential in nerve cells. As a result, the release of cytochrome C (Cytc) from the mitochondria is increased, and a polymerized complex of Cytc and apoptosis protein activating factor-1 (Apaf-1) is formed. This complex activates the precursor of caspase-9, which in turn activates downstream caspase-3, and thereby starts the apoptotic response cascade, leading to the ultimate loss of synapses and neuronal apoptosis [52,53,54]. B-cell lymphoma-2 (Bcl-2) and mitogen-activated protein kinase (MAPK) family member proteins have also been reported to play key roles in AD development [55,56,57].

### 2.2. Polyphenols Alleviate AD Symptoms by Reducing Oxidative Stress

Curcumin (1,7-bis (4-Hydroxy-3-Methoxy phenol) -1,6-heptadiene-3,5-dione) is a lipophilic polyphenol obtained from the rhizome of the Asian ginger plant Curcuma longa [58]. Due to its antioxidant [59], anti-inflammatory [60], antibacterial [61], anti-mutagenic [62], and anti-cancer [63] properties, curcumin is widely used in many areas, such as food spices, cosmetics, food pigments, and colorants. Curcuma longa L.—the source plant of curcumin—has long been used as part of herbal medicine practices in Asian countries as well [64]. Curcumin can directly scavenge free radicals (ROS and reactive nitrogen species, RNS). In vivo and in vitro experiments have also confirmed the role of curcumin in cell protection via activation of nuclear factor erythroid 2-related factor 2 (Nrf2)/Heme Oxygenase-1 (HO-1) and Nrf2/Kelch-like ECH-associated protein 1 (Keap1) pathways [65,66,67]. The amount of (Nrf2) in the brains of AD patients has been shown to be significantly lower than that in healthy individuals [68]. This signaling pathway plays a key role in Aβ deposition and thus delays the onset and development of AD. Curcumin was also shown to reduce copper-induced oxidative stress and mitochondrial apoptosis in SH-SY5Y cells and thereby improve neurotoxicity [69]. Fermented curcuma, including curcumin as its main component, was found to exert neuroprotective effects on C6 and BV2 cells against OS induced by H2O2 [70] and on rats against high intraocular pressure. Curcumin can provide neuroprotection by reducing ROS species and inhibiting the apoptosis pathway [71]. Curcumin was in vivo found to improve the overall behavior by increasing the CAT level in the brain of depression model mice [72]. Finally, curcumin and mitochondria-targeted curcumin (MTC) were found to have neuroprotective effects on a mouse model of cerebellar oxidative damage [73].

Resveratrol (3,4 ‘, 5-trihydroxystilbene), also known as stilbene triphenol, is a polyphenol that is abundant in grapes, pomegranates, mulberry, blueberries, and Reynoutria japonica Houtt [74]. Resveratrol was first isolated from Veratrum in 1939. The beneficial effects of resveratrol on human health have been emphasized for the first time through the “French paradox” (low risk of cardiovascular disease among French, despite their high-fat and -cholesterol diet), which is related to large amounts of daily red wine consumption among the French population [75,76]. Resveratrol has two isomers: cis-resveratrol and trans-resveratrol. Trans-resveratrol is the main form, and its effectiveness is conferred by the lower steric hindrance of its side chain [77]. Resveratrol has anti-inflammatory [78], antioxidative [79], anti-infective [80], antibacterial [81], and anti-cancer effects [82]. The main mechanisms involved with respect to the effect of resveratrol in the context of AD mainly involve antioxidant stress, anti-inflammatory effects, and Aβ aggregation [83].

OS markers that indicate the progression of AD were shown to appear earlier than pathological changes, including Aβ plaques and neurofibrillary tangles [84]. The antioxidative stress effect of resveratrol is mainly determined by its structure: 4’-OH and conjugated double bonds facilitate the transfer of hydrogen atoms or electrons to free radicals [85,86]. Resveratrol was also found to facilitate the production of free radicals and reactive oxygen species in BV2 mice, malondialdehyde production induced by rotenone, and the downregulation of glutathione synthesis by enhancing the inhibition of signal transducer and activator of transcription 1 (STAT1) and Keap1, and finally upregulation of the expression of Nrf2 and solute carrier family 7 (cationic amino acid transporter, y+ system) member 11 (SLC7A11) proteins. Hence, it plays a role in antioxidative stress as well [87]. Administration of resveratrol to c57bl/6 mice exposed to MnCl2 not only resulted in an improvement of the cognitive function of mice but also increased ROS levels and inhibited MDA activity significantly. On the other hand, SOD and CAT activities, L-Glutathione (GSH) content, and the ratio of GSH/ glutathione (GSSG) in the hippocampus were significantly increased [88].

Procyanidins (PC) are polyphenols that consist of flavanol monomers and their polymers and are abundant in all kinds of fruits, vegetables, bark, and peel, and especially in grapes and grape seeds, which was demonstrated to be a much stronger antioxidant than vitamin C or E [89,90,91,92]. The favorable pharmacological activities of procyanidins in terms of free radical scavenging [93], and antioxidative [94], anti-inflammatory [95], hypoglycemic [96], hypolipidemic [97], anticancer, and antitumor [98] effects have been confirmed by numerous studies. Neuroprotective effects of PC with different structures have also been recently proven. PC have thus been used to treat and prevent neurodegenerative diseases, including AD [99]. A unique tricyclic structure confers PC to its antioxidant capacity [100]. PC were found to significantly improve the cognitive ability of elderly rats, reduce increased protein oxidation and lipid peroxidation levels, enhance their antioxidant defense abilities, and significantly restore Ach activities [101]. In a mouse OS injury model induced by extremely low-frequency electromagnetic fields (ELF-EMF), administration of lotus seed pod proanthocyanidins was found to significantly enhance the activities of SOD, CAT, GPx, GR, and GST, and also reduced MDA levels, and thereby exerted neuroprotective effects [102]. The phenolic profiles of hardy kiwifruits rich in procyanidin B2 were also found to allow the reversal of oxidative stress in SH-SY5Y and PC-12 cells induced by H_2_O_2_ by improving their ability to resist acetyl cholinesterase (AchE) and butyrylcholinesterase (BchE) action [103]. Grape seed proanthocyanidins were found to improve the cognitive decline of mice caused by isoflurane and prevent OS by interfering with SOD activity and the NR2B/CREB pathway [104].

Tea is one of the most popular beverages worldwide and an important component of the East Asian diet. Although from the current clinical research, it seems uncertain to draw the conclusion that the consumption of tea, especially green tea, is significantly related to the improvement of cognitive impairment in AD patients. However, scientists do not deny that tea and its effective ingredients have certain neuroprotective effects [105,106,107]. Especially in animal experiments and in vitro experiments, tea has shown a better effect on improving cognitive function [108,109]. EGCG is a natural and the main active ingredient in green tea. ECCG has antioxidative [110], anti-inflammatory [111], anti-viral [112], cardioprotective [113], and anticancer effects [114,115]. In recent years, EGCG has also attracted the attention of researchers due to its neuroprotective effects, especially for the prevention and treatment of neurodegenerative diseases such as AD [116,117]. In one study, a daily intake of 400 mg/kg green tea polyphenols rich in EGCG by rats with chronic cerebral hypoperfusion was found to significantly improve cognitive function, eliminate free oxygen radicals, enhance antioxidative capacity, and reduce the production of lipid peroxide and oxidative DNA damage [118]. When rats with Streptozocin (STZ) injection into the ventricles were treated with EGCG, the levels of ROS and GSH levels in the hippocampus were found to decrease, and memory function was found to improve [119]. AD pathology is also related to brain iron imbalance and its association with amyloid precursor protein (APP) plaque formation. To this end, EGCG was found to increase the level of transferrin receptor (TfR) protein and mRNA in SH-SY5Y cells in a dose-dependent manner, and thereby play a role in iron chelation and reduce the expression of Swedish mutant APP in Chinese hamster oocytes [120].

**Figure 2:**
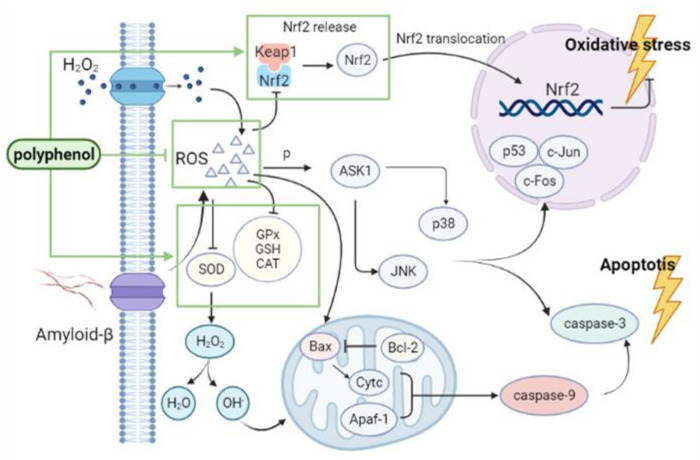
Regulatory roles of polyphenols in the context of oxidative stress: polyphenols can directly clear ROS and inhibit Keap1 to upregulate Nrf2 expression; increase SOD, CAT, and GSH activities; inhibit oxidative stress response; and thus alleviate AD symptoms. Nrf2, nuclear factor erythroid 2-related factor 2; Keap1, Kelch-like ECH-associated protein 1; p53, tumor protein P53; ROS, reactive oxygen species; ASK1, DASH complex subunit ASK1; SOD, superoxide dismutase; GPx, glutathione peroxidase; GSH, L-glutathione; CAT, catalase; Bax, apoptosis regulator BAX; Bcl-2, apoptosis regulator Bcl-2; Cytc, Cytochrome C; Apaf-1, apoptotic peptidase activating factor 1.

In general, the regulatory roles of polyphenols in the context of AD-related oxidative stress mechanisms are mainly based on the direct elimination of ROS; the regulation of SOD, CAT, and GSH levels; and the reduction of an oxidative stress injury in the brain through regulation of Nrf2/HO-1 and Nrf2/Keap1 pathways (Figure 2).

## 3. Polyphenols and Neuroinflammatory Mechanisms

### 3.1. Neuroinflammation in Alzheimer’s Disease

Due to the prevention of immune cells and humoral factors from entering the brain by the blood-brain barrier (BBB) and the inability of brain cells to produce an innate immune response, the brain was previously believed to enjoy immune privilege [121]. However, an increasing number of epidemiological observations and autopsy results refuted this theory by demonstrating that brain tissue can flexibly regulate its innate paracrine inflammatory factors [122,123,124]. The repeated failure of drugs that directly affect Aβ in clinical trials led some scientists to believe that direct regulation of Aβ is an unrealistic therapeutic intervention and that attention should instead be directed toward neuroinflammation mechanisms in AD [125].

Microglia, astrocytes, and monocytes play a significant role in the occurrence and development of inflammatory reactions in the central nervous system (CNS) [8,126]. Microglia can be considered immune cells of the brain since, although they account for only about 10% of the brain tissue, they constitute the first line of defenses against diseases [127,128]. Upon their activation, microglia in their normal resting state quickly travel to the vicinity of Aβ plaques and differentiate into pro-inflammatory (M1 microglia) and anti-inflammatory (M2 microglia) phenotypes that secret pro-inflammatory or anti-inflammatory cytokines, respectively. Pro-inflammatory cytokines activate microglia and astrocytes, aggravate nerve damage, decrease Aβ clearance, and ultimately aggravate the development of AD [129,130]. Astrocytes are the most abundant cells in the brain and perform various biological functions, such as blood flow regulation, maintenance of the BBB, supply of energy metabolites to neurons, regulation of synaptic activity, control of the secretion of neurotrophic factors, and removal of dead cells. Similar to microglia, astrocytes can also differentiate into two different phenotypes: pro-inflammatory (A1 astrocytes) and anti-inflammatory (A2 astrocytes). Both types of astrocytes perform their functions through the secretion of cytokines and the formation of cross-talk between microglia and coordinate their responses to regulate neuroinflammation [131,132,133,134]. At the same time, monocytes can also join in the “talk”, actively participate in the immune response and inflammatory response in the brain, and secrete a large amount of monocyte chemotactic protein 1 (MCP-1) after astrocytes are stimulated by Aβ, triggering the inflammatory cascade reaction by combining it with C-C motif chemokine receptor 2 (CCR2), and gradually differentiate into macrophages in the process of recruitment into the brain, playing the Aβ clearance ability as good as microglia [135,136,137]. The significant decrease in the phagocytosis of monocytes to Aβ in AD patients leads to the deposition of Aβ, which also indicates that monocytes play an important role in the anti-inflammatory treatment of AD [138,139]. Therefore, the development of targeted drugs to inhibit neuroinflammatory processes, such as the inhibition of microglia and astrocyte activation, and the enhancement of the phagocytic ability of monocytes/macrophages to Aβ is of great significance for the treatment of neurodegenerative diseases such as AD.

### 3.2. Polyphenols Can Alleviate AD Symptoms by Inhibiting Glial Inflammatory Activation, Affecting Monocyte/Macrophage System, and Inhibiting Neuroinflammation

The inflammatory response is a key factor in the development of most neurodegenerative diseases, such as Parkinson’s syndrome, multiple sclerosis, and AD [140]. Curcumin was previously found to promote the polarization of BV2 cells to the anti-inflammatory M2 phenotype through the triggering receptor expressed on myeloid cells-2 (TREM2)/Toll-like receptor 4 (TLR4)/nuclear factor kappa-B (NF-κB) pathway in vitro, and thereby inhibit lipopolysaccharide (LPS)-induced neuroinflammation [141]. The expression of matrix metalloproteinase-9 (MMP-9) and interleukin 6 (IL-6) secreted by human astrocytes (U373-MG) was found to be downregulated by curcumin after LPS induction, confirming the potential of curcumin to regulate astrocyte-mediated inflammation in the central nervous system [142]. Curcumin was also injected intraperitoneally into c57bl/6 mice in a distal middle cerebral artery occlusion (dMCAO) model to improve infarct area and motor sensory function, promote the expression of M2 polarization markers in microglia, and inhibit the expression of M1 polarization markers [143]. Reactive astrocytes in the brains of gfap-il6 mice treated with Meriva curcumin (MC) were also found to be significantly reduced. Hence, plant-based curcumin may indeed improve the pathology of neuritis by reversing the harmful effects of chronic glial cell activation [144]. In addition, it has been verified that curcuminoids can significantly improve the phagocytosis of macrophages to Aβ in AD patients [145,146].

The anti-inflammatory properties of resveratrol may be attributed to the inhibition of the production of anti-inflammatory factors. Resveratrol was found to inhibit the polarization of LPS-stimulated microglia to the pro-inflammatory phenotype M1 in vitro and in vivo through PGC-1α and promote the polarization of anti-inflammatory phenotype M2 through CO activation of signal transducer and activator of transcription 6 (STAT6) and signal transducer and activator of transcription 3 (STAT3), and thereby play an anti-inflammatory role [147]. The production of classical pro-inflammatory factors tumor necrosis factor-α (TNF-α), interleukin-1β (IL-1β), IL-6, and interleukin-18 (IL-18) released by primary astrocytes from the rat hippocampus and stimulated by LPS were also found to be inhibited by resveratrol. This anti-inflammatory effect is likely to be mediated through the regulation of NF-κB, HO-1, p38, and extracellular-signal-regulated kinases (ERK) pathways [148]. Resveratrol was also found to alleviate neuroinflammation in a rat astrocyte line (RA) and microglia (N9) caused by Aβ aggregation and plays an anti-inflammatory role by inhibiting NF-κB/p65 nuclear translocation [149]. In addition, resveratrol can reverse glutamate-induced upregulation of MCP-1 expression in rat hippocampus, reduce the release of inflammatory factor IL-1β, and alleviate neuroinflammation [150].

It was previously found that procyanidin dimers and trimers have significant anti-inflammatory activity [151,152,153,154]. Procyanidin A2 extracted from litchi seeds was found to significantly reduce the levels of TNF-α, IL-1β, and IL-6 released from the supernatant of BV2 neuroinflammatory model cells induced by Aβ1-42, and production levels of TNF-α, IL-1β, and inducible nitric oxide synthase (iNOS) by inhibiting the NF-B pathway to improve the inflammatory response [155]. Procyanidins were also found to inhibit LPS-induced p65 nuclear translocation and p38 MAPK phosphorylation in BV2 cells by regulating the p38 MAPK and NF-κB pathways. As a result, the inflammatory response is inhibited [156]. Proanthocyanidin treatment of mice with subcutaneous morphine-induced activation of spinal microglia attenuates chronic morphine tolerance by inhibiting the phosphorylation of NMDA-NR1, protein kinase C (PKC), and MAPKs in the spinal cord. Proanthocyanidin also inhibits the activation of microglia and NOD-like receptor thermal protein domain associated protein 3 (NLRP3) inflammasome in vitro and in vivo [157]. In addition, both procyanidin dimer B1 and trimer C1 can inhibit the inflammatory response signaling in human monocytes [153].

**Figure 3:**
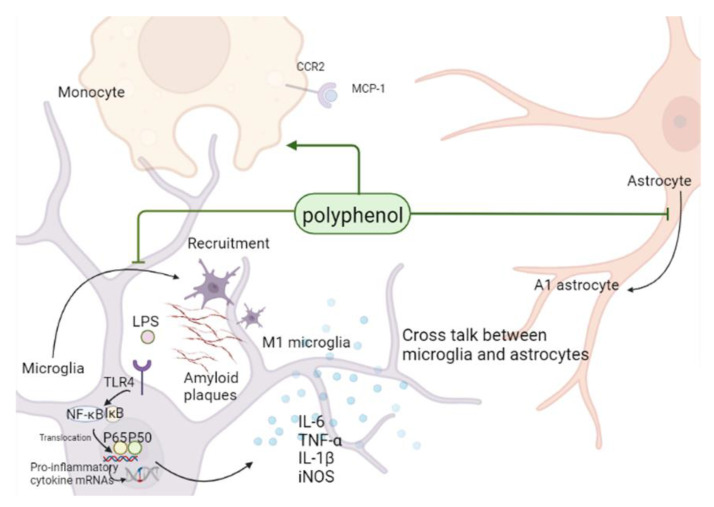
Regulatory roles of polyphenols in the context of neuroinflammation: polyphenols inhibit the activation of astrocytes and transformation of microglia to pro-inflammatory phenotypes in the brain by inhibiting NF-κB pathway, reduce the secretion of inflammatory factors, improve the inflammatory environment in the brain and affect the phagocytosis of monocytes/ macrophages, to eventually alleviate AD symptoms. LPS, lipopolysaccharide; NF-κB, tumor necrosis factor-α; TLR4, Toll-like receptor 4; IL-6, interleukin-6; IL-1β, interleukin-1β; TNF-α, tumor necrosis factor-α; iNOS, inducible nitric oxide synthase.

In conclusion, polyphenols inhibit the transformation of microglia and astrocytes to a pro-inflammatory phenotype and reactive astrocytes, respectively. Polyphenols further hinder the secretion of classical inflammatory factors such as TNF-α, IL-1β, and IL-6 by regulating the NF-κB pathway to improve inflammatory cross-talk in the brain and affecting monocyte/macrophage system to alleviate the symptoms of AD (Figure 3).

## 4. Polyphenols and Amyloid Toxicity Mechanism

### 4.1. Amyloid Neurotoxicity in Alzheimer’s Disease

As the core of the amyloid hypothesis was first proposed in 1992, Aβ denotes short peptides composed of approximately 36–43 amino acids that are produced through hydrolysis of APP by β- and γ-secretory enzymes [158,159]. Amyloid plaque deposition in the brain as a result of the gradual aggregation and accumulation of Aβ is a major pathological feature of AD and has been recognized by scientists as the main cause of AD for a long time [160]. Aβ production is closely related to the single transmembrane protein APP, which is mainly located in the brain. Mutations in the APP gene locus and presenilin 1 and 2 (PSEN-1, -2) result in abnormal upregulation of beta-secretase 1 (BACE-1) and γ-site cutting enzyme activities. The resulting erroneous cleavage leads to the production and accumulation of excessive Aβ peptides. These peptides show toxic effects on axons and synapses; lead to neuronal degeneration, necrosis, and synaptic dysfunction; and eventually induce pathological changes [161,162]. However, an increasing number of researchers have started to question the relevance of this hypothesis to AD development. To this end, the connection between Aβ accumulation and AD development is not considered a simple causal relationship and may be explained by accelerated assembly of AD-like polymers, which in turn damages cholinergic neurons, and leads to excessive accumulation and activation of microglia to induce neuroinflammation. This, in turn, affects mitochondrial-dominated energy metabolism, leading to oxidative stress and abnormal phosphorylation of tau protein, creating a more complex feedback cascade that promotes the symptoms of AD [134,163,164,165,166,167].

### 4.2. Regulatory Role of Polyphenols in the Context of Amyloid Neurotoxicity

As mentioned above, the erroneous cleavage of β- and γ-secretase enzymes will eventually lead to large amounts of Aβ production, and the Aβ that cannot be cleared in time will deposit in the brain and eventually form toxic amyloid deposits, resulting in AD pathology. Therefore, in the current study, the relationship between polyphenols, Alzheimer’s disease, and amyloid toxicity hypothesis can be summarized as follows: polyphenols inhibit the production of Aβ by affecting the activity of secreted enzymes or inhibit the self-assembly of amyloid into fibrils by relying on their own aromatic rings with hydroxyl groups, and promote the depolymerization and clearance of Aβ [168,169]. In this way, the pathological phenomena of AD can be alleviated to a certain extent.

The effect of resveratrol on BACE1 and γ-secretase may occur via two distinct mechanisms. Nine resveratrol oligomers extracted from peony were found to inhibit BACE-1 expressed in baculovirus in a dose-dependent manner [170]. Resveratrol derivatives extracted from Vitis vinifera were also found to inhibit BACE-1 expression by baculovirus in a dose-dependent manner [171]. Resveratrol and its analogs were also found to decrease γ-secretase and increase α-secretase activities in Neuro2a neuroblastoma cells [172]. However, some studies have reported that resveratrol only facilitates the reduction of the Aβ content in cells and has, in fact, no effect on the α-, β-, or γ-secretase-mediated cleavage of APP or the stability of its C-terminal fragment. Resveratrol was also shown not to affect APP metabolism and Aβ production. The therapeutic potential of resveratrol for AD may therefore be based on its proteasome-dependent anti-amyloidosis activity [173]. The focus of research on the effect of resveratrol in AD thus shifted to Aβ clearance mechanisms such as activation of Aβ degradation enzymes [174], modulation of the plasminogen system [175], maintenance of the integrity of the BBB, and modulation of the expression of transporters and receptors to regulate the steady-state levels of Aβ [176].

Many clinical studies showed the central role of Aβ-polymerized fibrils or other self-assembly states in AD pathogenesis [177]. Procyanidins show their potential for AD treatment by affecting Aβ accumulation, which is manifested through the following mechanisms. First, the aggregation of Aβ is inhibited: several type-A procyanidins and procyanidins from apple were found to inhibit the aggregation of Aβ fibrils and show depolymerization activity as observed by experimental observations based on transmission electron microscopy (TEM) or thioflavin-T (Th-T) fluorescence staining [178,179]. This may be related to the fact that the catechol moiety is easily oxidized automatically and covalently bound to the nucleophilic amino acid residues of amyloid protein, rendering the structure of the β-fold unstable [180,181,182]. This may also be related to the presence of multiple hydroxyl groups in procyanidins themselves, which can be related to the strong interaction between amino acids that hinders the formation of β-fold forms [183]. Second, the particle size distribution of peptide aggregates is affected. Upon intervention by procyanidins, the particle size of Aβ decreases from the micron to the nanometer range. Third, the morphology of the peptide aggregates is modified. Following co-incubation with procyanidins, the fibrils of the Aβ peptide become smaller spherical particles. Finally, peptide depolymerization is promoted, and thereby mature fibrils are turned into shorter fibrils [184].

As with any other polyphenol, EGCG also inhibits the aggregation of Aβ fibrils in a dose-dependent manner [185]. Comparative evaluations of the capacities of several common polyphenols to inhibit Aβ aggregation showed that EGCG is a significantly stronger inhibitor of Aβ aggregation than other polyphenols, including curcumin and resveratrol [186]. The underlying mechanism may involve changes in the conformation of Aβ into an Aβ assembly without the “seeding” ability [187]. The aromatic ring of EGCG may also prevent the C-terminal of Aβ from obtaining a β-fold conformation. Thus, the oligomer structure is gradually stabilized and cannot form fibrils further [188]. Oral administration of EGCG (50 mg/kg) to APP/PS1 transgenic AD model mice for 4 months was found to significantly alleviate cognitive deficits in mice, improve dendritic integrity and synaptic protein expression levels in the brain, significantly reduce Aβ plaques in the hippocampus of mice, and alleviate the neuroinflammatory response by reducing microglial activation, inhibiting the secretion of pro-inflammatory cytokines IL-1β, and increasing the secretion of anti-inflammatory cytokines interleukin-10 (IL-10) and interleukin-13 (IL-13) [189]. EGCG was also found to increase α-secretase and reduce β- and γ-secretase activities by acting through the ERK/NF-κB pathway, thereby reducing the production of Aβ aggregates [190,191].

**Figure 4:**
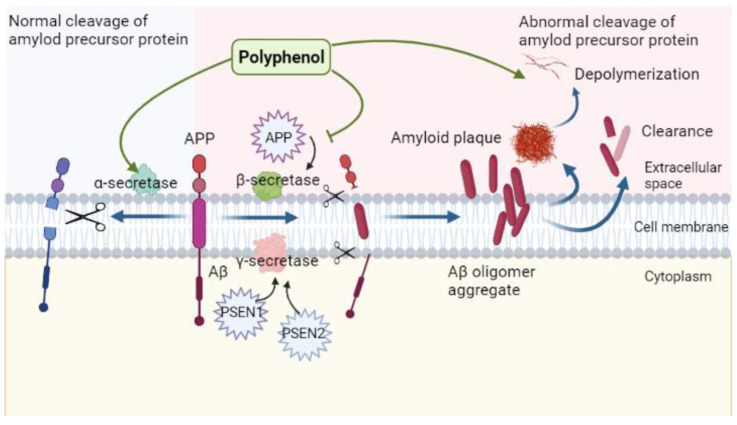
Regulatory role of polyphenols in the context of amyloid neurotoxicity: polyphenols allow cleavage of Aβ correctly by promoting the activity of α-secretase, inhibit the activities of β-, and γ-secretases, prevent erroneous cleavage, promote Aβ plaque depolymerization and clearance, and reduces its neurotoxicity.

In conclusion, the inhibitory effect of polyphenols on Aβ aggregation is mainly based on the inhibition of Aβ production by affecting the activities of α-, β-, and γ-secretory enzymes, promoting the clearance, and depolymerization by affecting the conformation of Aβ fibrils, and reduction of neurotoxicity (Figure 4).

## 5. Polyphenols and Abnormal Tau Protein Phosphorylation

### 5.1. Abnormal Phosphorylation of Tau Protein in Alzheimer’s Disease

The tau protein hypothesis is mainly based on the abnormal deposition of neuronal tangles, a pathological phenomenon in AD [192]. The tau protein is soluble under normal conditions and regulates the stabilities of microproteins. However, its over-phosphorylation renders the protein insoluble, and the phosphorylated tau competes with binding to normal tau protein. This then leads to the depolymerization of microtubules and the formation of paired helices in neurons, thereby disrupting the normal physiological function of the tau protein [2,193,194]. As a marker of neuronal injury, the content of total tau protein in the cerebrospinal fluid of AD patients is higher than that in normal individuals. The proportion of abnormally phosphorylated tau protein is significantly increased as well [195]. Similar to the Aβ plaque deposition, abnormal phosphorylation of tau can also not be detected in the early stages of AD, yet previous findings indicate that the abnormal accumulation of tau is a better indicator of AD onset time [3]. Therefore, the current AD treatment methods based on tau protein are mainly divided into non-immunotherapeutic approaches based on stabilizing microtubules, inhibition of tau protein phosphorylation and aggregation, or inhibition of pathological tau diffusion, and active and passive immunotherapy approaches, including vaccination and monoclonal antibodies. Non-immunotherapeutic approaches are, in general, ineffective [196,197,198].

### 5.2. Regulatory Role of Polyphenols in the Context of Abnormal Tau Protein Phosphorylation

The role of curcumin in improving tau-based AD pathology has been preliminarily investigated [199]. Curcumin administration to human Tau (hTau) transgenic mice exhibiting the tau pathology was found to reduce the level of soluble tau dimers, reverse the interruption of the expression of molecular chaperones heat-shock protein 90 (HSP90), heat-shock protein 70 (HSP70), and heat-shock protein 72 (HSP72) in hTau expressing mice, and improve spatial learning and memory abilities [200]. Curcumin was also found to inhibit tau oligomerization by preventing β-folding, which is the initial step of tau aggregation, and decomposing the formed tau protein filaments in vitro [201]. Curcumin treatment of transgenic Caenorhabditis elegans with tau-induced neuronal dysfunction did not significantly affect tau aggregation, yet improved neuron functions and stabilized microtubules [202]. EGCG interacts with full-length tau protein via multiple residues to destabilize its structure and dissolve tau fibrils and oligomers while inhibiting the aggregation of full-length tau. Thus, the tau protein remains mostly in the unfolded monomer state [203,204]. EGCG was also found to enhance the clearance of abnormally phosphorylated tau in primary neurons by enhancing connexin expression in an in vitro model [205]. Oral administration of EGCG (50 mg/kg) to APPsw mice for 6 months significantly improved their cognitive ability and reduced the expression of hyperphosphorylated tau in the brain [206].

## 6. Influence of Polyphenol on Cholinergic Injury

### 6.1. Cholinergic Injury in Alzheimer’s Disease

The cholinergic injury hypothesis is a widely recognized mechanism of AD [207]. The significant loss in basal forebrain cholinergic neurons has attracted the attention of scientists as an early pathological feature of AD [208]. Previous findings revealed defects in the central cholinergic system responsible for regulating learning and memory in AD patients. One of the main reasons for this defect is the abnormal enhancement of AchE activity [209]. AchE inhibitors can therefore protect neurons by reversibly binding to the enzyme, thereby preventing acetylcholine hydrolysis, increasing the level of acetylcholine in the brain, and eventually enhancing cholinergic transmission. Indeed, drugs designed to inhibit AchE activity, such as donepezil, tacrine, and galantamine, have been widely used for AD treatment to date [210].

### 6.2. Regulatory Role of Polyphenols in the Context of Cholinergic Injury

**Figure 5:**
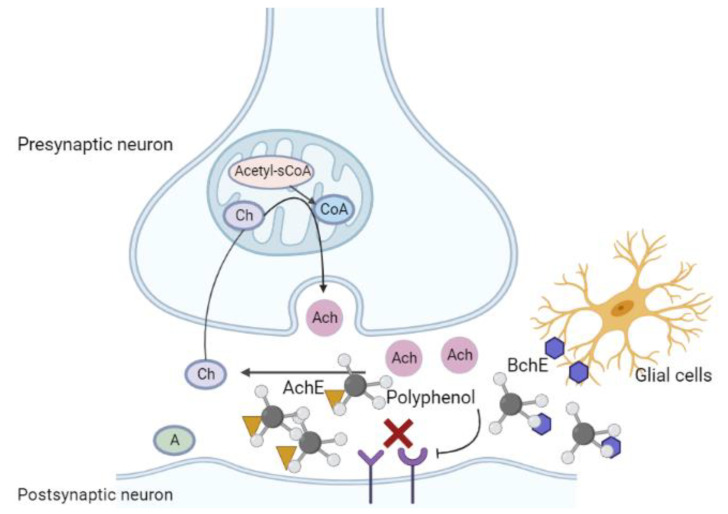
Regulatory role of polyphenols in the context of cholinergic injury: polyphenols inhibit the binding of cholinesterase to receptors, reduce cholinesterase activity, reduce Ach hydrolysis, restore the cholinergic level in the brain, and alleviate AD symptoms. Ach, acetylcholine; AchE, acetyl cholinesterase; BchE, butyrylcholinesterase.

Polyphenols mainly inhibit the binding of cholinesterase to receptors, reduce its activity, and thus reduce the hydrolysis of Ach to restore cholinergic levels, improve cognitive ability, and alleviate AD symptoms (Figure 5). Curcumin has been reported to regulate the function of the cholinergic system and improve the levels of diminished nutritional factors in neurodegenerative diseases. Following the administration of curcumin to cadmium-treated (neurotoxic) rats, AchE activity and gene expression in the cerebral cortex were found to be significantly reduced, confirming the inhibitory effect of curcumin on AchE activity in the nervous system in vivo [211]. Curcumin can also inhibit AchE activity in the hippocampus and cortex of rats with dementia induced by intracerebroventricular (ICV) injection of STZ [212].

EGCG was also shown to have an inhibitory effect on AchE. A comparative evaluation of five natural flavane-3-alcohol (catechin) compounds isolated from green tea using the Ellman method: (-)—epicatechin (EC), catechin, (-)—epicatechin-3-gallate (ECG), (-)—epigallocatechin (EGC), and (-)—epigallocatechin-3-gallate (EGCG) revealed that only EGCG significantly inhibited AchE [213]. Oral administration of EGCG to rats with chronic brain dysfunction induced by an ICV injection of STZ reduced AchE activity in the hippocampus as well [119]. Other studies have further confirmed that the oral administration of EGCG (2 mg/kg/day, lasting for 30 days) increases the level of Ach in the cerebral cortex of aged rats [214]. Moreover, molecular docking simulations have also confirmed the interaction between green tea polyphenols through combined effects on AchE and BChE [215].

## 7. Polyphenols and ApoE in Alzheimer’s Disease

### 7.1. ApoE Gene in Alzheimer’s Disease

ApoE and its three isoforms, ApoE2, ApoE3, and ApoE4, play significant roles in plasma cholesterol metabolism, atherosclerosis, and hypercholesterolemia [216,217,218,219,220]. ApoE was also shown to be the main apolipoprotein lipid and cholesterol transporter in the central nervous system and is produced by nearly all nerve cell types, including astrocytes in the physiological state, microglia in the pathological state, and neurons following specific injuries [221,222,223,224]. AD is also considered by some a genetic disease caused by mutations in several genes, including ApoE. ApoE4—the ε4 allele of ApoE—has attracted attention as the strongest risk gene since it is most commonly found in patients with late-onset AD [224,225,226]. ApoE4 is associated with several pathological features of AD. For example, patients carrying this gene have more obvious speech memory and cognitive impairment, and Aβ plaque deposition and pathological tau protein phosphorylation occur earlier than in other AD patients [227,228]. ApoE4 not only affects Aβ-related mechanisms of AD [229,230,231,232,233] but also aggravates Tau-mediated neurodegeneration [234,235] and even promotes the expression of inflammatory cytokines. Hence, carriers of this gene develop a stronger systemic inflammatory response [236]. Although the exact mechanism is not yet clear, it can be argued that the ApoE gene hypothesis is closely related to other hypotheses of AD development.

### 7.2. Regulatory Role of Polyphenols in the Context of ApoE Gene

Our knowledge of polyphenols regulating ApoE gene function is mainly based on studies of atherosclerotic diseases. Only several studies have focused on the regulation of the ApoE gene by polyphenols in AD models [237]. Researchers have previously found that the secretion of inflammatory factors is reduced upon administration of the extract of Arabidopsis seedlings, which increases the yield of polyphenolsin mixed glial cell cultures extracted from the cerebral cortex of ApoE-targeted replacement (ApoE TR) mice or ApoE knockout (ApoE KO) mice. This suggests that polyphenols inhibit ApoE-derived neuroinflammation [238]. Other studies have confirmed that single polar phenolic compounds can improve the changes in cell redox state and cytotoxicity after induction and that kaempferol can regulate the pathogenic conformation of the ApoE4 form by providing ApoE-induced SK-N-SH cell blackcurrant extract (whose main components are resveratrol, quercetin, kaempferol, and epigallocatechin gallate) [239].

## 8. Polyphenols and Other Mechanisms in Alzheimer’s Disease Development

### 8.1. Polyphenols and Insulin Resistance

Most patients with AD develop many other complications as well, such as obesity, stroke, and type 2 diabetes mellitus (T2DM). These complications may coordinate or aggravate the development of AD [240]. A meta-analysis revealed that diabetic patients are more likely to convert from mild cognitive impairment (MCI) to AD compared to non-diabetic AD patients, which indicates that T2DM increases the vulnerability of patients to neurodegenerative diseases, including AD [241]. T2DM has been proven to be associated with AD through insulin resistance [242]. Insulin resistance refers to the decline in the efficiency of insulin in promoting glucose uptake and utilization and the compensatory secretion of excessive insulin by the body to produce hyperinsulinemia, which is the main cause and pathological phenomenon of T2DM [243]. Polyphenols were reported to effectively inhibit insulin resistance. For example, insulin resistance in APP/PS1 transgenic mice was found to decrease via upregulation of the PI3K/Akt signaling pathway upon curcumin treatment [244]. Resveratrol and EGCG were also found to enhance the activity of insulin-degrading enzyme (IDE) to decompose extracellular Aβ in neurons, and promote its clearance in hyperinsulinemia caused by insulin resistance [245,246].

### 8.2. Polyphenols and Mitochondrial Dysfunction

Mitochondrial dysfunction is one of the most important mechanisms in the early stages of AD, which is mainly caused by oxidative phosphorylation induced by insufficient glucose metabolism. Ultimately, dysfunctional mitochondria produce less ATP and more ROS. This, in turn, leads to pathological changes in AD, such as Aβ deposition, formation of NFTs, and synaptic degeneration [247]. The recombinant sirtuin (SIRT) family plays an important role in regulating mitochondrial proteins to maintain homeostasis during oxidative stress and apoptosis. Recombinant sirtuin 3 (SIRT3) has been proposed as a new target for AD treatment [248]. Previous studies have revealed that in the AD model presenilin (psev1-v97l) transgenic mice honokiol upregulates the expression of mitochondrial SIRT3, increases the amount of produced ATP, decreases the amount of produced ROS, improves the cognitive ability of mice, and thereby alleviates AD symptoms [249]. Other researchers found that urolithin A (UA), a type of ellagitannin found in pomegranate and walnuts, reduces mitochondrial membrane potential (MMP) and adenosine triphosphate (ATP) levels in a cellular model of early AD and increases the expression of genes involved in mitochondrial biogenesis and mitochondrial respiration (oxidative phosphorylation, OXPHOS). Urolithin A may thus also show therapeutic effects on AD [250].

### 8.3. Polyphenols and Faulty Autolysosome Acidification

The pathogenesis of AD has traditionally been accepted to originate from the formation of Aβ amyloid plaques. Here, amyloid plaques are first formed and then cause a series of toxic reactions to kill nerve cells. However, according to this concept, clearing plaques cannot remedy dead nerve cells according to this mechanism. This raises the question of whether Aβ plaques are the cause or effect. A recent study where a variety of AD mouse models were used disproved this traditional hypothesis and instead proposed that the occurrence of AD begins with the death of nerve cells caused by faulty autolysosome acidification of nerve cells, followed by the formation of extracellular amyloid plaques [251]. The hydrolases in lysosomes are only active in acidic environments, which are mainly maintained by the proton pump V-ATPase that pumps H+ ions from the cytoplasm into the lysosomes. Once lysosomal acidification is blocked, the pipeline of substrate removal stops, resulting in the formation and accumulation of many waste intermediates in lysosomes, including Aβ and APP. When the lysosome cannot bear rupture, the hydrolase is released into the cytoplasm to decompose the digestive cells and disintegrate them, eventually forming “extracellular” amyloid plaques [251,252]. Therefore, the main idea of treatment based on this mechanism is to restore the acidification function of the lysosomes. Previous experiments have shown that resveratrol activates adenosine 5‘-monophosphate (AMP)-activated protein kinase (AMPK) through pro-autophagic mechanisms and directly interacts with SIRT1 in vitro to induce autophagy by controlling SIRT1-mediated transcriptional regulation or mTOR-dependent signaling pathways. Resveratrol thus shows the potential to treat AD through these mechanisms [253,254,255,256]. Other studies have also revealed that EGCG resists incomplete autophagy induced by the hepatitis B virus (HBV) and inhibits its replication by enhancing lysosomal acidification [257]. Apple polyphenol extract (APE) has also been proven to restore lysosomal acidification by activating the SIRT1/AMPK signaling pathway, which may indicate another strategy for AD treatment [258].

### 8.4. Polyphenols and Disruption of the Intestinal Flora

The two-way communication system along the flora-gut-brain axis includes neural, immune, endocrine, and metabolic pathways, all of which have a profound impact on brain function and cognitive ability [259]. The relative abundance of bacterial species in patients with cognitive impairment was reported to change in previous studies. This change is manifested by an increase in the abundance of pro-inflammatory Escherichia coli/Shigella species and a decrease in the abundance of anti-inflammatory Escherichia coli [260]. On the other hand, the proportion of beneficial bacteria that synthesizes short-chain fatty acids (SCFA: acetate, propionate, butyrate) was found to decrease in the fecal flora of AD patients, whereas the abundance of pro-inflammatory bacteria was found to increase [261]. This suggests that the loss of intestinal homeostasis and inflammation caused by disruption of the intestinal flora may lead to the development of AD. Previous studies have revealed the effect of polyphenols on the richness, diversity, and composition of intestinal microbiota [262]. Curcumin was also found to improve the memory and cognitive abilities of APP/PS1 transgenic mice, increase intestinal bacterial diversity and reduce the abundance of Escherichia coli/Shigella [263]. In addition, a resveratrol-selenium-peptide nanocomposite was also found to improve cognitive impairment in AD model mice by reducing intestinal bacterial flora disruption due to oxidative stress and inflammation, such as Alistipes, Helicobacter pylori, Rikenella, Desulfovibrio, and Faecalibaculum [264]. The idea of treating AD by regulating intestinal flora has received increasing attention in the clinic, yet more research is still needed to confirm the proposed mechanisms.

## 9. Potential Regulation Effect of Polyphenols on Recently Identified Targets of AD

Mammalian STE20-like protein kinase (MST) is an important component of the Hippo pathway, in which MST1/2 regulates neuronal death and neuroinflammation [265,266]. A recent study revealed that Mst1 activity is increased with the accumulation of Aβ in the hippocampus of 5xfad mice. The overexpression of MST1/2 was also found to induce an AD-like phenotype in normal mice yet without a significant effect on Aβ levels. This was found to accelerate the decline in cognitive abilities of 2-month-old mice and caused damage to synaptic plasticity. It was also found to promote neuronal apoptosis by activating and phosphorylating p53. Knockout or chemical inactivation of Mst1 was found to be effective in alleviating AD symptoms [267]. Mst1 is therefore expected to become a potential target for the treatment of AD. Although there are currently only a few reports on the treatment of AD with polyphenols with this target, studies have already shown that tea polyphenols reverse the upregulation of MST1/2 in RAW264.7 cells after H_2_O_2_ application, inhibit oxidative stress through the MST/Nrf2 axis, and Keap1/Nrf2/HO-1 signaling pathways, and reduce the production of ROS, the release of NO, and the level of MDA in cells [268]. These findings highlight the therapeutic potential of polyphenols in alleviating AD symptoms by acting on this target.

Receptor-interacting protein kinase 1 (RIPK1), as a kind of serine/threonine protein kinase, a member of the RIP family, has been widely mentioned in research related to necrosis [269,270,271]. It was found that RIPK1 was highly expressed in AD patients, AD model mice, and microglia of the AD model [272]. In addition, RIPK1 inhibitors can reduce the levels of Aβ and p-tau in the cortex and hippocampus of APP/PS1 mice [273]. These data indicate that RIPK1 is expected to become a potential target for the treatment of AD. Although there are currently only a few reports aimed at this target to treat AD with polyphenols, some studies have shown that curcumin can reduce the levels of oxidative stress biomarkers and inflammatory cytokines and reduce the necrosis and apoptosis of chicken liver tissue by regulating TLR4/RIPK signal pathway [274]. In addition, some studies have also confirmed that resveratrol can inhibit myocardial cell necrosis and apoptosis by inhibiting TNF-a/receptor-interacting protein kinase 1 (RIP1)/RIP3/mixed-lineage kinase domain-like (MLKL) signaling pathway [275]. This suggests that the possible therapeutic ability of polyphenols on AD can be studied from this target in the future.

## 10. The Potential and Mechanisms of Action of Other Common Plant Polyphenols for AD Treatment

Polyphenols are widely found in various common plant species and show unlimited potential for screening of active ingredients for the prevention and treatment of AD. As mentioned above, polyphenols can alleviate AD symptoms through several mechanisms, including antioxidant stress, reduction of neuroinflammation, inhibition of Aβ production and aggregation, and inhibition of AchE activity. Curcumin, resveratrol, proanthocyanidins, and EGCG are extensively studied plant polyphenols in terms of their potential for the prevention and treatment of AD. However, the use of other plant polyphenols for AD treatment has also gradually attracted attention (Table 2).

Previous studies have emphasized that in aging-related neurodegenerative diseases, including AD, plant polyphenols do not just affect a specific mechanism of the pathogenesis of these diseases, such as inhibition of Aβ production and aggregation, anti-inflammatory response, and antioxidant stress. The therapeutic effect on AD may also alleviate disease symptoms by regulating the links between multiple targets and mechanisms [304]. Some polyphenols were found to produce rapid benefits after a single administration, especially in terms of attention function and working memory, yet these polyphenols need to be consumed over a long period of time if adequate observations are to be made. Researchers also prefer to administer polyphenols in the early stage of the disease, based on the expectation that dietary intake of polyphenols is more suitable for the early stage of AD [43,305]. In this stage, polyphenols mainly play a therapeutic role in antioxidant stress, anti-inflammatory activity, inhibition of Aβ production and aggregation, and inhibition of AchE activity.

## 11. Limitations in the Application and Improvements in Preparation of Polyphenols

An increasing number of studies have revealed that a diet rich in polyphenols can prevent the onset of AD. Therefore, interest in easily obtained plant polyphenols with low toxicity has increased recently. Hence, the therapeutic potential of these compounds against AD has also been studied by more scientists over time. However, polyphenols also suffer from limitations that hinder their clinical use, such as low bioavailability and their unclear role in the BBB [306,307].

Although curcumin shows a certain degree of neuroprotective effect due to its strong hydrophobicity and low solubility, it has poor absorption and stability at physiological pH and suffers from rapid systemic elimination, poor bioavailability, and low brain bioavailability due to the BBB. The retention time of curcumin in the brain is very short [307,308,309]. To overcome this problem, different curcumin carriers, such as liposome curcumin, curcumin nanoparticles, and curcumin phospholipid complexes, have been developed previously [310]. Curcumin-loaded solid lipid nanoparticles (SLN) and nanostructured lipid carriers (NLC) were found to improve the stability of curcumin and increase its permeability through the BBB by a factor of 1.5 [311]. The melting crystallization method was also found to effectively improve the solubility of curcumin and wrap it into the cavity of lipid poly (lactic-co-glycolic acid) (PLGA) nanobubbles. The resulting curcumin-loaded lipid PLGA nanobubbles (Cur NBS) can pass through the BBB and deliver the drug to the brain core, which was found to significantly alleviate the symptoms in PD mice [312].

Similarly, the clinical use of resveratrol is also limited owing to its low bioavailability. Extensive metabolism in the gut and liver leads to an oral bioavailability of less than 1%, and dose escalation and repeated administration were found to be ineffective [313]. Since the success of the treatment of nervous system diseases largely depends on the bioavailability of drugs, novel strategies are needed for the use of resveratrol in a clinical context [314]. Currently, resveratrol delivery nanocarriers developed for the treatment of AD mainly include lipid core nano capsules (NC), polymer micelles, and SLN [315]. A comparison of resveratrol-loaded NC and free resveratrol in rats exposed to Aβ showed that resveratrol-loaded NC can play a neuroprotective role by increasing the concentration of resveratrol in brain tissue and reducing the harmful effects caused by Aβ, such as memory loss, learning difficulties, synaptophysin level reduction, and astrocyte and microglial activation [316]. Polymer micelles loaded with resveratrol can reduce the cytotoxicity of free resveratrol, protect PC12 cells from Aβ through antioxidant stress, and reduce the activity of Caspase-3 [317].

Although proanthocyanidins can promote health, only monomers and smaller oligomeric forms of proanthocyanidins can be absorbed. Proanthocyanidins with a degree of polymerization of more than four cannot be absorbed due to their large molecular sizes and the presence of the intestinal barrier [318]. High clearance and rapid metabolism of proanthocyanidins after oral administration were found to result in a significantly lower bioavailability (8–11%), as estimated from the blood area under the concentration–time curve (AUC) (0–24) values [319]. The absorption and metabolism of procyanidin B2 following oral administration in mice were also studied, and a total of 45 metabolites of procyanidin B2 were identified. The small intestine included the highest proportion of metabolites compared to other tissues. The liver was also found as the main binding reaction organ for procyanidin B2. Furthermore, procyanidins are among the few polyphenols that can penetrate the blood-brain barrier after oral absorption. The metabolites of procyanidin B2 in the mouse brain include hydration, sulfonation, and dihydrate products [320]. The optimization of the dosage form of procyanidins mostly involves the production of microcapsules, nano-emulsions, liposomes, and clathrate compounds in order to improve stability and bioavailability simultaneously [321].

Polyphenols have considerable application potential in the field of natural products as drugs and health products owing to their antioxidant, antibacterial, anti-inflammatory, and other favorable biological activities [322]. However, most polyphenols are insoluble and unstable, and their low absorption and bioavailability limit their clinical and industrial application [323]. With the development of improved nanomaterials for biomedical applications, polyphenol-loaded drug delivery systems that can improve the in vitro stability and bioavailability of polyphenols have been favored by researchers [324]. For example, polymer nanoparticles, lipid-based nanocarriers, gold–silver nanoparticles, silica nanoparticles of resveratrol, lipid nanoparticles, protein nanoparticles, and PLGA nanoparticles of EGCG have been developed in recent years [325].

## 12. Conclusions

**Figure 6:**
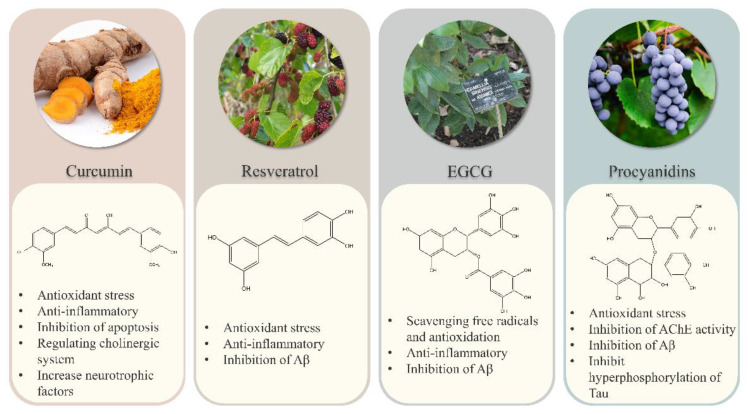
Mechanisms of action of curcumin, resveratrol, proanthocyanidins, and EGCG in AD treatment: Curcumin, resveratrol, proanthocyanidins, and EGCG alleviate the pathological symptoms of AD mainly through antioxidant stress, anti-neuroinflammation, inhibition of Aβ production and inhibition of AchE.

In conclusion, recent scientific evidence shows that the development of AD is accompanied by pathological changes such as oxidative stress, Aβ deposition, abnormal phosphorylation of tau protein, neuroinflammation, and abnormal acetylcholine activity, in addition to changes in various other physiological mechanisms. Due to the complex molecular mechanisms related to AD, prevention and treatment can not only target a single protein or gene but also need to consider new strategies for multiple proteins and genes. Polyphenols, as secondary metabolites widely found in natural plants, especially curcumin, resveratrol, proanthocyanidins, EGCG, and other plant polyphenols, can alleviate the symptoms of AD patients through antioxidative, anti-inflammatory, Aβ inhibitory, and other neuroprotective effects when used as dietary supplements (Figure 6).

Future research on polyphenols should focus on clinical applications, and especially the connection between in vitro and animal experiments. If the bioavailabilities of several powerful polyphenols need to be further improved, the preparation of nanoparticles, liposomes, and other new dosage forms that do not affect function while maintaining high bioavailability should be prioritized in clinical research. Moreover, it should also be recognized that in vitro AD experiments cannot completely simulate the long course of a disease that lasts for several years. Therefore, more long-term experiments and large-scale epidemiological and clinical studies are needed to determine whether polyphenols have sufficient beneficial effects on the slow development of AD. The combined use of several widely studied polyphenols is likely to have a greater effect. In addition, it is also necessary to evaluate the risks and safety of the use of polyphenols and investigate the possible adverse reactions to medication in clinical research. In general, the development of plant polyphenols with low toxicity and high availability as drugs has good potential to prevent and alleviate AD symptoms.

## Figures and Tables

**Table 1:** The mechanisms of action and adverse reactions of drugs approved by Food and Drug Administration (FDA) for Alzheimer’s disease (AD) treatment.

Drugs	Mechanisms of Action	Main Limitations	Reference
Tacrine	Inhibition of acetylcholinesterase activity and increase in acetylcholine level	Oral administration leads to strong hepatotoxicity and gastrointestinal adverse reactions, which quickly leads to increase in transaminase activity	[11,12]
Donepezil	Inhibition of acetylcholinesterase activity and increase in acetylcholine level. Inhibition of aberrant glia cell activation to alleviate neuroinflammation	Low CNS selectivity, gastrointestinal toxicity (nausea, vomiting, anorexia, flatulence, loose stool, diarrhea, salivation, and abdominal colic)	[13,14,15]
Rivastigmine	Selective enhancement of Ach activity in the cerebral cortex and hippocampus. Improvements in cognitive function and deceleration of APP formation	Adverse reactions such as acute dystonia, nausea, vomiting, diarrhea, dizziness, and weight loss	[16,17,18]
Galantamine	Inhibition of acetylcholinesterase activity and increase in acetylcholine level. Regulation of nicotinic acid receptors outside the brain to increase Ach release	Severe cutaneous adverse drug reactions	[19]
Memantine	Antagonizing effect on NMDAR	Bradycardia	[20,21]
Aducanumab	Recognition of an epitope of Aβ, reduction of aggregated soluble and insoluble forms of Aβ.	ARIA, effusion, minor hemorrhage, and hemosiderosis	[22]

CNS, central nervous system; Ach, acetylcholine; APP, amyloid precursor protein; NMDAR, N-methyl-D-aspartate-receptor; ARIA, amyloid-associated imaging abnormality.

**Table 2:** Prevention and treatment potential of polyphenols on AD and its mechanism.

Classification	Compounds	Main Sources	AD Model	Dose and Time	Mechanism	Reference
Flavone	Apigenin	Apium graveolens, Petroselinum crispum, Citrus sinensis, Vitis vinifera, Allium sativum, Marchantia polymorpha	APP/PS1 mice	40 mg/kg, 12 weeks	Antioxidant stress and inhibition of Aβ production	[276]
			Aβ_1–42_ or LPS-induced coculture of cortical neurons and glial cells in neonatal Wistar rats	1 μM, 24 h	Anti-inflammatory	[277]
	Baicalin	Scutellaria baicalensis Georgi	APP/PS1 mice; Aβ_1–42_-induced BV2 and SH-SY5Y cells	100 mg/kg, 33 days; 0, 10, 20, 40 µM, 24 h	Inhibiting NLRP3 inflammatory corpuscle activation by TLR4/NF-κB pathway	[278]
	Kaempferol	Allium tuberosum, Allium cepa, Vigna radiata, Cucurbita moschata, Solanum tuberosum, Solanum lycopersicum, Fragaria ananassa, Forsythia suspensa, Rosmarinus officinalis, Acacia farnesiana, Ginkgo, Mimosa pudica, Cinnamomum tamala	Female ovariectomized (OVX) Wistar rats induced by ICV of STZ	10 mg/kg, 21 days	Antioxidative stress, anti-neuroinflammation	[279]
			Lipoprotein particles containing ApoE4-induced SK-N-SH cells	20 μM, 24 h	Changing the conformation and function of ApoE4	[239]
	Luteolin	Arachis hypogaea, Brassica napus, Olea europaea, Fagopyrum esculentum, Theobroma, Capsicum annuum, Apium graveolens, Daucus carota, Cucumis sativus, Lactuca sativus, Punica granatum	3 × Tg-AD mice	20, 40 mg/kg, 3 weeks	inhibiting endoplasmic reticulum stress-dependent neuroinflammation	[280]
Flavanol	Epicatechin	Tea-leaves, cocoa, grape seeds, Fagopyrum dibotrys, Amygdalus persica, Malus pumila	Methamphetamine (METH) induced HT22 cells	10, 20 μM, 1 h	Antioxidant	[281]
	Myricetin	Fragaria ananassa, Malus pumila, Spinacia oleracea, Aloe vera, Daucus carota, Fructus Mori, red wine	Primary rat cortical neurons induced by Aβ_1–42_	10μM, 48 h	Inhibiting the activity of BACE-1; inhibiting Aβ	[282]
	Quercetin	Citrus reticulata, Momordica charantia, Malus pumila, Allium cepa, Vitis vinifera, Vaccinium spp, Rubus idaeus	Aβ_25–35_-induced PC-12 cells	10, 20, 40, 80 μM, 48 h	Antioxidant	[283]
			Okadaic acid (OA)-induced HT22 cells	5, 10 μM, 12 h	Antioxidant, inhibiting tau hyperphosphorylation	[284]
			APP/PS1 mice	2 mg/kg, 7–8 months	inhibition of Aβ production	[285]
Phenolic acid	Caffeic acid	Solanum lycopersicum, Daucus carota, Fragaria ananassa, Semen Trigonellae, wheat wine, tea, coffee, apple juice	Mice induced by ICV of Aβ_25–35_	10, 50 mg/kg, 14 days	Antioxidant	[286]
	Gallic acid	Vitis vinifera, Lilium brownii viridulum, Punica granatum, Rosa rugosa, Toxicodendron vernicifluum, Quercus palustris, Hamamelis mollis, Rhus chinensis, Terminalia chebula	APP/PS1 mice	20 mg/kg, 6 months	Antioxidant stress, anti-inflammatory, affecting α-, β-, γ-secretase activity	[287]
			AlCl3-induced Wistar rats	100 mg/kg, 60 days	Antioxidant	[288]
			APP/PS1 mice	30 mg/kg, 30 days	Inhibiting Aβ_1–42_ aggregation	[289]
			Aβ_1–42_-induced primary microglia and BV2 cells	5–50 μM, 24 h	Inhibiting NF-κB acetylation, antioxidant	[290]
Phenylpropanoid	Chlorogenic acid	coffee, Lycium chinense, Lonicera japonica, Eucommia ulmoides, Arctium lappa, Chrysanthemum indicum, Malus pumila, Prunus pseudocerasus, Camellia sinensis	APP/PS1 mice; Aβ_25–35_-induced SH-SY5Y cells	40 mg/kg, 6 months; 3.125, 6.25, 12.5, 25, 50 µM, 24, 48 h	Inhibiting excessive autophagy and Aβ production by regulating mTOR/TFEB signaling pathway	[291]
			Aβ_25–35_-induced primary rat hippocampal neurons	12.5, 25, 50 μM, 2 h	Antioxidant	[292]
	Ferulic acid	Triticum sativum, beer, coffee, berries, Avena sativa, Ananas comosus, Arachis hypogaea	APP/PS1 mice	20 mg/kg, 30 days	Improving hippocampal capillary perfusion insufficiency	[293]
			ICR mice induced by ICV of Aβ_1–42_	14–19 mg/kg, 30 days	Antioxidant	[294]
Flavanonol	Dihydromyricetin	Vitis vinifera, Myrica rubra, Ampelopsis grossedentata, Ginkgo	SD rats induced by ICV of Aβ_1–42_	100, 200 mg/kg, 21 days	Anti-inflammatory by activating AMPK/SIRT1 signaling pathway	[295]
			LPS-induced BV2 and primary microglias	10, 30, 50 μM, 1 h	Anti-inflammatory by NF-κB signaling pathway	[296]
Isoflavone	Genistein	Glycine max	Wistar rats induced by bilateral ICV of Aβ_1–42_	10 mg/kg, 10 days	Attenuating synaptic toxicity, inhibiting tau hyperphosphorylation, inactivating ERK	[297]
			Wistar rats induced by ICV of STZ	150 mg/kg, 30, 90 days	Anti-inflammatory; Stimulating autophagy of Aβ_40, 42_ and p-tau	[298]
			Aβ_25–35_-induced SH-SY5Y cells	1, 10 nM, 24 h	Inhibiting Aβ induced apoptosis and Akt inactivation, inhibiting tau hyperphosphorylation	[299]
Flavonone	Hesperidin	Citrus reticulata Blanco	C57BL/6N induced by ICV of Aβ_1–42_; Aβ_1–42_-induced BV2 and HT22 cells	50 mg/kg, 6 weeks; 50 μM, 24 h	Inhibiting oxidative stress by regulating Nrf2/HO-1; Inhibiting neuroinflammation by regulating TLR4/NF-κB; Inhibiting the expression of APP, BACE-1, Aβ	[300]
Ellagitannin	Punicalagin	Punica granatum	LPS-induced ICR; Primary rat cortical astrocytes and BV2	1.5 mg/kg, 4 weeks; 10, 20, 50 μM, 24 h	Anti-inflammatory	[301]
Isothiocyanate	Sulforaphane	Brassica oleracea, Nasturtium officinale, Brassica oleracea var. acephala DC, Brassica oleracea var. capitata, Brussels sprouts, Brassica rapa var. glabra Regel, Brassica juncea, Brassica oleracea var. botrytis Linnaeus	5 × FAD mice	5, 10 mg/kg, 2 months	Inhibiting Aβ, inhibiting phosphorylation tau	[302]
Benzotropolones	Theaflavin	Camellia sinensis	ICR mice induced by ICV of LPS; LPS, IFN-γ induced primary microglias	10, 50 mg/kg, 3 days; 10, 30 μM, 12 h	Anti-inflammatory	[303]

## Data Availability

Not applicable.
